# Thrombospondin Type-1 Repeat Domain-Containing Proteins Are Strongly Expressed in the Head Region of *Hydra*

**DOI:** 10.1371/journal.pone.0151823

**Published:** 2016-04-04

**Authors:** Kayoko Hamaguchi-Hamada, Mami Kurumata-Shigeto, Sumiko Minobe, Nozomi Fukuoka, Manami Sato, Miyuki Matsufuji, Osamu Koizumi, Shun Hamada

**Affiliations:** 1 Department of Food and Health Sciences, International College of Arts and Sciences, Fukuoka Women’s University, Fukuoka, Japan; 2 Department of Environmental Sciences, International College of Arts and Sciences, Fukuoka Women’s University, Fukuoka, Japan; UC Irvine, UNITED STATES

## Abstract

The head region of *Hydra*, the hypostome, is a key body part for developmental control and the nervous system. We herein examined genes specifically expressed in the head region of *Hydra oligactis* using suppression subtractive hybridization (SSH) cloning. A total of 1414 subtracted clones were sequenced and found to be derived from at least 540 different genes by BLASTN analyses. Approximately 25% of the subtracted clones had sequences encoding thrombospondin type-1 repeat (TSR) domains, and were derived from 17 genes. We identified 11 TSR domain-containing genes among the top 36 genes that were the most frequently detected in our SSH library. Whole-mount *in situ* hybridization analyses confirmed that at least 13 out of 17 TSR domain-containing genes were expressed in the hypostome of *Hydra oligactis*. The prominent expression of TSR domain-containing genes suggests that these genes play significant roles in the hypostome of *Hydra oligactis*.

## Introduction

**Fig 1:**
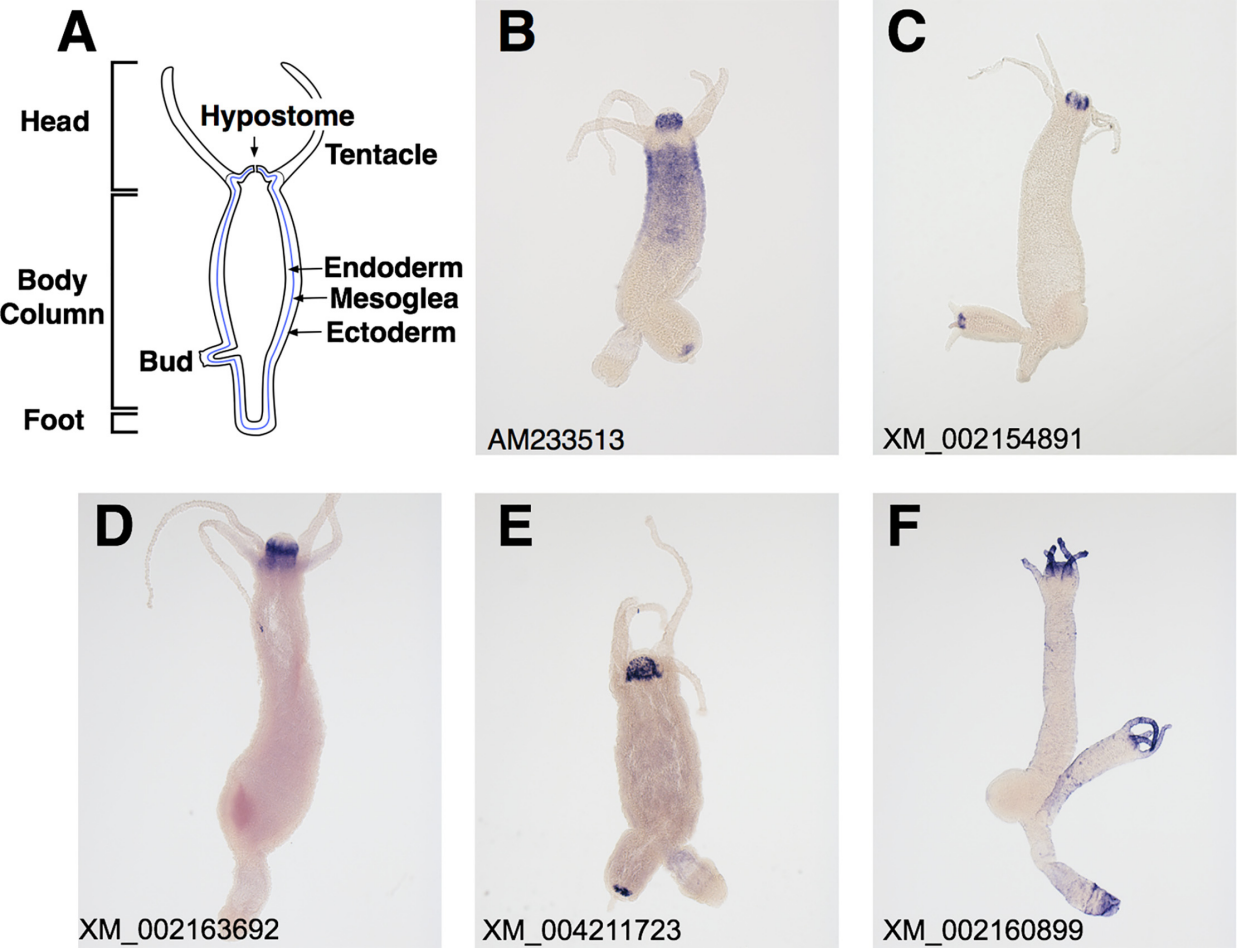
Expression patterns of the top five genes encoding TSR domain proteins in *Hydra oligactis*. (A) A schematic drawing of a longitudinal cross section of an adult hydra. (B-F) Expression patterns of the top five genes encoding TSR domain proteins in *Hydra oligactis* by whole mount *in situ* hybridization. All the genes are expressed in the head region. The accession numbers of the genes were shown on the lower left in each panel.

*Hydra*, a member of the phylum Cnidaria, has a simple diploblastic body plan with an oral-aboral axis including a head surrounded by tentacles, a body column, and a foot (Fig 1A). The tissue of adult *Hydra* is always in a steady state of production and loss. Thus, axial patterning processes are continuously active in adult *Hydra* [1, 2]. *Hydra* also exhibits strong regeneration capabilities, and, thus, serves as a model organism for understanding the establishment and maintenance of the axial pattern and cell fate [3].

The hypostome of *Hydra* (Fig 1A), the region around the mouth above the tentacle bases, is a key body part for developmental control [4] and the nervous system [5, 6]. The hypostome has been identified as an organizer that controls axial patterning processes in *Hydra*. Previous studies reported that an isolated piece of the hypostome induced the formation of a secondary axis when it was transplanted into the body column of another *Hydra* [7, 8]. The head organizer in the hypostome is thought to be established and maintained by the canonical Wnt pathway [9–12], which appears to be involved in establishing the organizer in other animals [4]. Although the mechanisms by which the head organizer induces the secondary axis currently remain unclear, diffusible signals produced by the organizer are proposed to be involved in this process [13]. Thus, the morphogenetic molecules expressed in the hypostome need to be examined in more detail.

In addition to the role of the organizer, the hypostome is a unique part in the *Hydra* nervous system. The basic design of the *Hydra* nervous system is a nerve net [14], which comprises diffusely distributed neurons connected to each other without the formation of distinct nerve tracts. The hypostome contains the highest density of neurons in the nerve net [15, 16]. Furthermore, some hydra species including *Hydra oligactis* possess a nerve tract, the nerve ring, in the hypostome [5, 6, 17]. The hypostome may function as the pacemaker for rhythmic contractions of the body column of *Hydra* [18]. These findings suggest that the nerve net in the hypostome has some of the characteristics of the centralization of the nervous system [19].

The importance of the hypostome in the development and nervous system of *Hydra* prompted us to examine genes specifically expressed in this region. In the present study, suppression subtractive hybridization (SSH) [20] was used to identify head region-specific genes in *Hydra oligactis*. Although several studies using SSH have been performed on *Hydra* (regenerating tissue [21], budding tissue [22], interstitial cells [23], immunostimulated tissue [24, 25], developmental stages [26], and species-specific genes [27]), region-specific genes have not yet been investigated in *Hydra*.

The DNA sequences of the cDNA clones obtained by SSH were used to search for homology in the nucleotide, protein, and conserved domain databases. Of the clones obtained by our SSH procedure, 25% showed homology to genes containing the thrombospondin type-1 repeat (TSR) domain, many of which are considered to play roles in cell-cell and cell-extracellular matrix interactions [28, 29].

## Materials and Methods

### Animals and culture conditions

*Hydra oligactis* was cultured in hydra culture medium (1 mM NaCl, 1 mM CaCl_2_, 0.1 mM MgSO_4_, 1 mM Tris, pH7.6) at 19°C and fed 6 days a week with hatched *Artemia* cysts. Experiments were performed after a 2-day starvation period.

### RNA extraction

Total RNA was extracted from tissues using TRI® Reagent (Invitrogen, Carlsbad, CA, USA). In order to obtain head region tissues, we cut animals (approximately 200 polyps) just below the base of the tentacles, retained a piece of the tissue around the mouth, and then removed the tentacles. The body region tissue was a piece of the body just adjacent to the head region, the size of which was similar to the head region tissue.

### SSH and cDNA library construction

Tester cDNA was generated from 1 μg of total RNA from the head region tissues using the SMART cDNA synthesis kit (Clontech, Takara Bio Co., CA, USA) according to the manufacturer’s instructions. We used 1 μg of total RNA from the body region tissues for driver cDNA.

SSH was performed using the PCR-Select cDNA Subtraction Kit (Clontech) according to the manufacturer’s protocol. The resulting subtracted cDNAs were cloned into the pBluescript II KS+ vector (Agilent Technologies, CA, USA). Following blue/white selection, white DH5α *E*. *coli* clones were examined by colony hybridization with driver cDNA as the probe in order to remove transformants that contained cDNAs for the mRNA expressed in the body region. Clones showing no hybridization signal were individually selected and grown in medium. Plasmid isolation and the determination of cDNA sequences were conducted according to standard procedures.

### Sequence analysis and conserved domain searching

A homology search for the DNA sequences of 1414 clones was performed on the National Center for Biotechnology Information (NCBI) nucleotide database using the Basic Local Alignment Search Tool nucleotide program (BLASTN). The Conserved Domain Database (CDD) search (CD-search) at the NCBI site [30] was used for the protein domain analysis of the predicted amino acid sequences translated from the DNA sequences of the clones.

### Whole mount *in situ* hybridization (WISH)

The protocol originally described by Grens et al. (1996) [31] was used for all *in situ* hybridization experiments. Digoxigenin (dig)-labeled cRNA probes were generated by *in vitro* transcription using T3 or T7 RNA polymerase with PCR-amplified cDNA clones as templates. Hydras were relaxed with 2% urethane and fixed in 4% paraformaldehyde at 4°C overnight. After fixation, fixed animals were dehydrated and rehydrated in a graded series of methanol diluted with PBS containing 0.1% Tween 20 (PBT). After the proteinase K treatment and acetylation, the tissues were refixed in 4% paraformaldehyde for 30 min. Tissues were rinsed and then heated to 80°C in order to eliminate endogenous alkaline phosphatase (AP) activity. Hybridization was performed with dig-labeled cRNA probes (0.4μg /ml) in the hybridization solution (HS) containing 50% formamide, 5 x SSC, 0.1% CHAPS, 0.02% yeast tRNA, 1 x Denhardt’s solution, 0.01% heparin, and 0.1% Tween 20 at 55°C for approximately 60 hr. After hybridization, tissues were washed with HS at 55°C, 75% HS-25% 2 x SSC, 50% HS–50% 2 x SSC, and 25% HS–75% 2 x SSC for 10 min each, and then twice with 2 x SSC with 0.1% CHAPS for 30 min. The hybridized probes were detected by AP-conjugated anti-dig Fab fragments (Roche, Nutley, NJ, USA) diluted 1:2000 in maleate buffer (80 mM maleic acid and 120 mM NaCl, pH7.5) containing 20% sheep serum. Excess amounts of the antibody were removed by washing with maleate buffer. Hybridization signals were visualized with BCIP and NBT substrate solution (Roche, Nutley, NJ, USA) at 4°C in complete darkness for approximately 10 min ~ 2 hr. Stained tissues were dehydrated with ethanol and mounted in Euparal (Asco Laboratories, Manchester, England).

## Results

In order to identify genes selectively expressed in the hypostome of *Hydra oligactis*, we constructed a subtractive cDNA library containing head-specific genes by SSH. We determined the DNA sequences of 1414 subtracted clones, and then analyzed the sequences obtained using a local BLASTN program (E-value < 1.0e-10). Fourteen percent (195 clones) of the analyzed clones showed no similarity to any sequence in the NCBI nucleotide database (nr-nt). The remaining 1219 clones were grouped into 540 genes based on the results of the BLASTN search. Table 1 lists the nucleotide database records in descending order of the number of clones (at least four clones) that had nearly identical nucleotide sequences to the same database record (Data acquisition date: 04 August 2014). The remaining records that have two or three nearly identical nucleotide sequences are listed in S1 Table.

**Table 1:** Results of a BLAST homology search of cDNA clones isolated from the SSH library of *Hydra oligactis*.

N	Gene name (best BLASTN match with E-value <1e-10)	Accession number ^a^	Number of clones ^b^	E-value ^c^
**1**	***Hydra magnipapillata* mRNA for thrombospondin type 1 repeat-containing protein 2 precursor (tsrp2 gene)**	**AM233513**	**157**	**0.0E+00**
**2**	**PREDICTED: *Hydra magnipapillata* uncharacterized LOC100198704 (LOC100198704), partial mRNA**	**XM_002154891**	**56**	**0.0E+00**
3	PREDICTED: *Hydra magnipapillata* heparanase-like (LOC100212115), mRNA	XM_002170704	54	0.0E+00
4	PREDICTED: *Hydra magnipapillata* uncharacterized LOC100205717 (LOC100205717), mRNA	XM_002157156	31	7.5E-82
**5**	**PREDICTED: *Hydra magnipapillata* uncharacterized LOC100198541 (LOC100198541), mRNA**	**XM_002163692**	**29**	**0.0E+00**
**6**	**PREDICTED: *Hydra magnipapillata* SCO-spondin-like (LOC100210677), partial mRNA**	**XM_004211723**	**28**	**0.0E+00**
7	PREDICTED: *Hydra magnipapillata* uncharacterized LOC100212279 (LOC100212279), mRNA	XM_002163024	23	4.5E-170
8	PREDICTED: *Hydra magnipapillata* uncharacterized LOC100203064 (LOC100203064), mRNA	XM_002163545	21	1.3E-65
9	PREDICTED: *Hydra magnipapillata* seed linoleate 9S-lipoxygenase-3-like (LOC100213504), mRNA	XM_002167228	21	0.0E+00
**10**	**PREDICTED: *Hydra magnipapillata* uncharacterized LOC100202129 (LOC100202129), mRNA**	**XM_002160899**	**17**	**2.8E-153**
**11**	**PREDICTED: *Hydra magnipapillata* collagen alpha-4(VI) chain-like (LOC100213391), partial mRNA**	**XM_002170479**	**17**	**0.0E+00**
**12**	**PREDICTED: *Hydra magnipapillata* SCO-spondin-like (LOC101236385), partial mRNA**	**XM_004208316**	**16**	**2.8E-177**
13	PREDICTED: *Hydra magnipapillata* uncharacterized LOC100197067 (LOC100197067), mRNA	XM_002157271	12	1.1E-155
14	PREDICTED: *Hydra magnipapillata* uncharacterized LOC100215883 (LOC100215883), mRNA	XM_002162974	11	1.7E-155
**15**	**PREDICTED: *Hydra magnipapillata* uncharacterized LOC101238626 (LOC101238626), mRNA**	**XM_004205753**	**10**	**2.7E-172**
16	PREDICTED: *Hydra magnipapillata* transmembrane protein 175-like (LOC100206560), mRNA	XM_002160340	9	0.0E+00
17	PREDICTED: *Hydra magnipapillata* zinc metalloproteinase nas-6-like (LOC100197494), mRNA	XM_002157361	8	0.0E+00
**18**	**PREDICTED: *Hydra magnipapillata* uncharacterized LOC100201155 (LOC100201155), mRNA**	**XM_002168659**	**8**	**4.7E-47**
19	PREDICTED: *Hydra magnipapillata* heparanase-like (LOC100208368), partial mRNA	XM_002166527	7	3.3E-86
20	PREDICTED: *Hydra magnipapillata* uncharacterized LOC100215810 (LOC100215810), mRNA	XM_004206591	7	0.0E+00
21	PREDICTED: *Hydra magnipapillata* brachyury protein-like (HyBra2) (LOC100204831), mRNA	XM_002153980	6	1.8E-173
22	PREDICTED: *Hydra magnipapillata* uncharacterized LOC100204023 (LOC100204023), mRNA	XM_002158609	6	2.6E-119
23	PREDICTED: *Hydra magnipapillata* chymotrypsinogen B-like (LOC100213885), mRNA	XM_002159056	6	5.4E-169
**24**	**PREDICTED: *Hydra magnipapillata* uncharacterized LOC100200589 (LOC100200589), partial mRNA**	**XM_002159679**	**6**	**3.5E-115**
25	PREDICTED: *Hydra magnipapillata* uncharacterized LOC100212432 (LOC100212432), partial mRNA	XM_002167642	6	8.3E-14
26	*Hydra oligactis* cyclic GMP-dependent protein kinase (hyGK) mRNA, complete cds	AF031931	5	0.0E+00
27	*Mus musculu*s targeted KO-first, conditional ready, lacZ-tagged mutant allele Slco6b1:tm1a(KOMP)Wtsi tm1a(KOMP)Ucd tm2a(KOMP)Ucd tm2a(KOMP)Wtsi; transgenic	JN949777	5	2.9E-57
28	PREDICTED: *Hydra magnipapillata* actin, non-muscle 6.2-like (LOC100206987), mRNA	XM_002154660	5	0.0E+00
29	PREDICTED: *Hydra magnipapillata* tubulin beta-4 chain-like, transcript variant 1 (LOC100214668), mRNA	XM_002161877	5	4.1E-109
30	PREDICTED: *Hydra magnipapillata* actin, non-muscle 6.2-like (LOC100202036), mRNA	XM_002154426	4	0.0E+00
31	PREDICTED: *Hydra magnipapillata* uncharacterized LOC100214198 (LOC100214198), mRNA	XM_002154441	4	7.5E-175
32	PREDICTED: *Hydra magnipapillata* golgi-associated plant pathogenesis-related protein 1-like (LOC100212373), mRNA	XM_002154698	4	0.0E+00
**33**	**PREDICTED: *Hydra magnipapillata* uncharacterized LOC100208689 (LOC100208689), mRNA**	**XM_002154766**	**4**	**2.6E-113**
34	PREDICTED: *Hydra magnipapillata* uncharacterized LOC100215554 (LOC100215554), mRNA	XM_002159136	4	2.6E-39
35	PREDICTED: *Hydra magnipapillata* secreted signaling factor Wnt1 (hywnt1), mRNA	XM_002162939	4	0.0E+00
36	PREDICTED: *Hydra magnipapillata* uncharacterized LOC100202525 (LOC100202525), mRNA	XM_002165570	4	7.6E-169

^a^ Accession numbers were annotated according to the NCBI database.

^b^ The number of sequenced clones in the SSH library.

^c^ The best e-value from a BLASTN search.

Most of these nucleotide database records are predicted cDNA sequences of *Hydra magnipapillata*, the genome of which has been sequenced and analyzed [32]. In these records, we found predicted cDNA sequences for *Hydra magnipapillata* Wnt1 (No.35) and Wnt7 (No.41). In addition to Wnts, we found a predicted cDNA sequence for *Hydra magnipapillata* brachyury-like protein (HyBra2, No.21), which is expressed in the hypostome, possibly under the control of the Wnt pathway [33]. Furthermore, a predicted cDNA sequence for Frizzled (No.83), a component of Wnt receptors, was included in the records.

We next searched the CDD (CD-search) using the deduced amino acid sequences of the nucleotide database records. We found that 11 out of the top 36 records in Table 1 (the rows in bold) contained sequences encoding the TSR domain. Furthermore, 358 clones (25.3%) of the examined 1414 clones had sequences encoding TSR domain proteins. This result indicates that the genes encoding TSR domain proteins are strongly expressed in the head. The results of our WISH analyses revealed that the top 5 genes for TSR domain proteins (AM233513, XM_002154891, XM_002163692, XM_004211723, XM_002160899) were strongly expressed in the head region (Fig 1B–1F).

**Fig 2:**
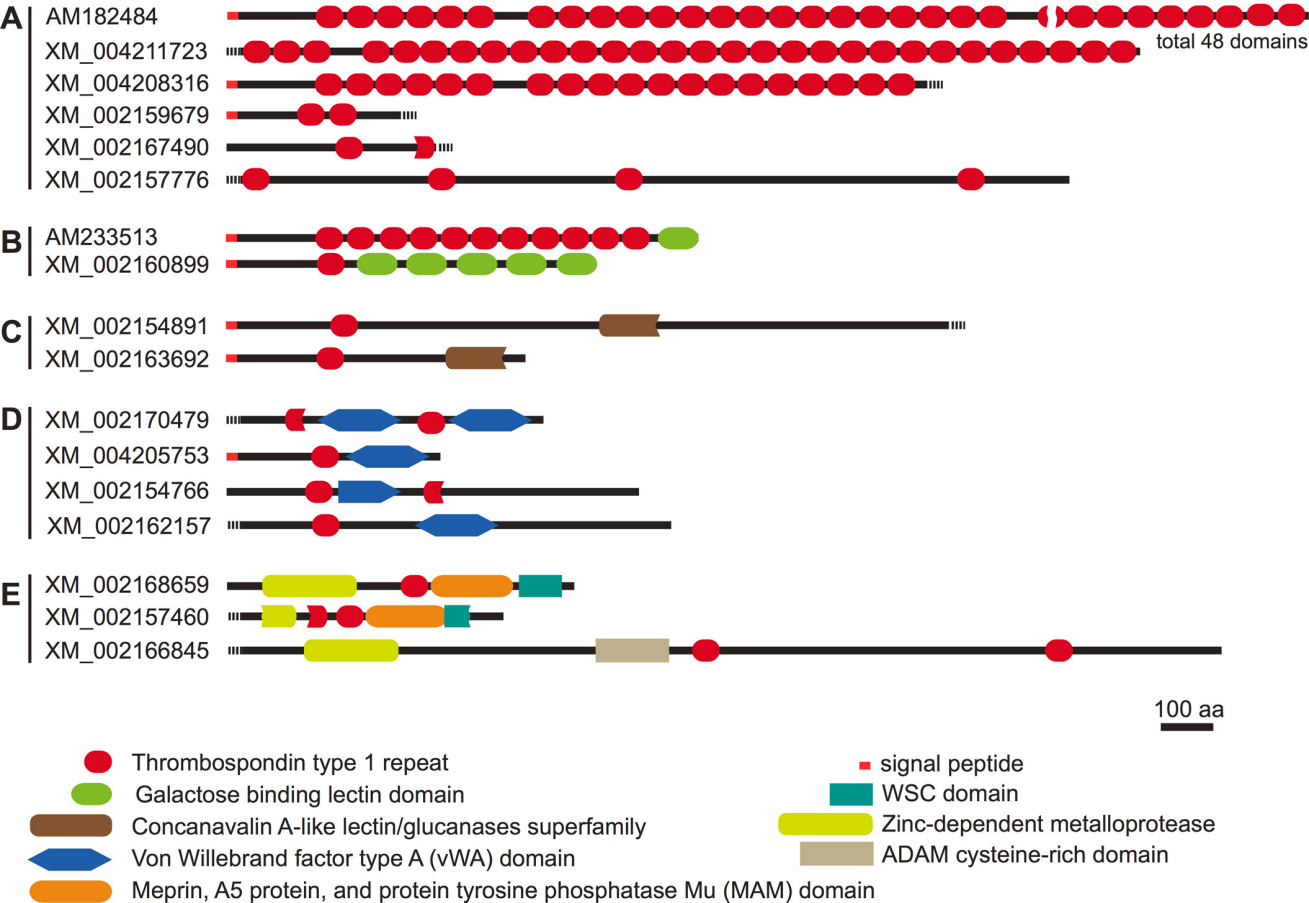
Domain structures of *Hydra magnipapillata* TSR domain proteins. The domain structures of TSR domain proteins identified in the present study. TSR domains are indicated by red ovals. The accession numbers are from the NCBI nucleotide database.

Our BLASTN analyses revealed that 358 cDNA clones encoding TSR domain proteins were derived from 17 genes. We tentatively classified these genes into five groups according to the domain structures (Fig 2), even though most of these genes, excluding *HyTSR1* (AM182484) and TSR-containing protein 2 precursor (*Tsrp2*, AM233513), were predicted.

**Fig 3:**
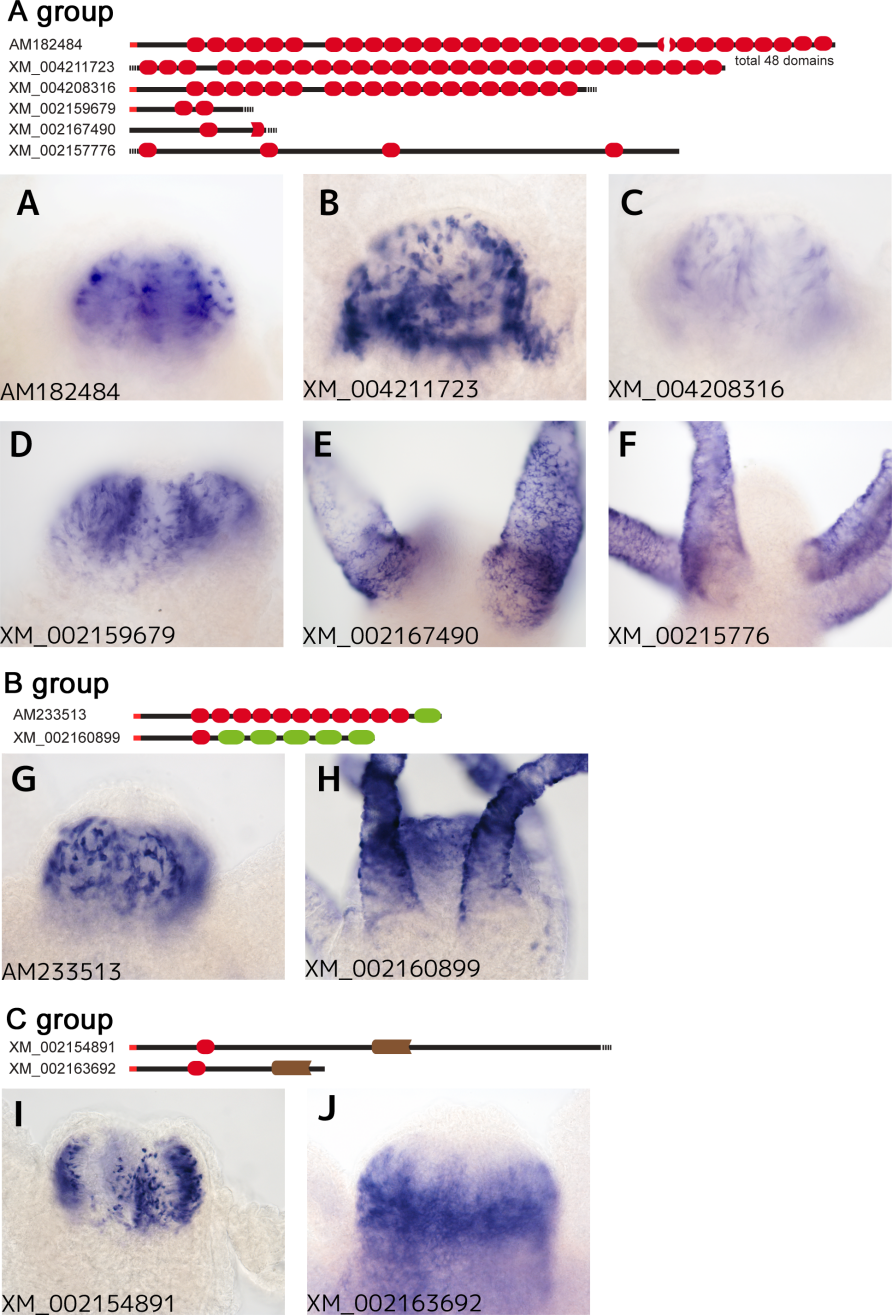
Expression patterns of the genes encoding TSR domain proteins of the A-C groups in the head region of *Hydra oligactis*. Among the 10 genes of the A-C groups, six (A- D, G and I) are strongly expressed in the endodermal cells and two (H and J) in the ectodermal cells of the hypostome. The transcripts of XM_002167490 (E) and XM_00215776 (F) were only detected in the tentacles, and not in the hypostome. XM_002160899 (H) was detected in the ectodermal cells of both the hypostome and tentacles. Accession numbers were labeled on the lower left of each panel. The domain structures of the genes were shown on the top of photo panels for each group.

The first group (Fig 2A) comprised six genes containing multiple TSR domains with no other predicted conserved domains. Among these genes, *HyTSR1*, which contains 48 TSR domains, was previously reported to be exclusively expressed in the hypostome, specifically in granular mucous gland cells [34] (Fig 3A). Since the other genes in this group were all predicted and have not yet been analyzed, we examined the expression patterns of the other members of this group in *Hydra oligactis*. XM_004211723 contained at least 28 TSR domains, and was intensely expressed in the endoderm cells of the head region, most likely in non-epithelial cells, possibly gland mucous cells or neurons (Figs 1E and 3B). XM_004208316 and XM_002159679 were also expressed in non-epithelial cells of the endoderm; however, their expression levels varied markedly (Fig 3C and 3D). In contrast, XM_002167490 and XM_00215776 mRNA were detected in the tentacles, particularly in the epithelial cells of the ectoderm (Fig 3E and 3F).

The second group was characterized by the existence of the galactose-binding lectin domain and TSR domain (Fig 2B). We identified two genes as members of this group. One of the two genes, the TSR-containing protein 2 precursor (*Tsrp2*, AM233513) gene, had previously been cloned and analyzed [35]. *Tsrp2* was composed of 11 TSR domains and a single galactose-binding lectin domain. The cDNA clones for *Tsrp2* were the most abundant among the clones obtained by our SSH procedure (156 clones of 1414 cDNA clones, Table 1). *Tsrp2* mRNA was strongly expressed in the non-epithelial cells of the endoderm in the hypostome (Figs 1B and 3G). In addition to the hypostome, the upper region of the body column also expressed *Tsrp2* mRNA (Fig 1B). The other gene, XM_002160899 was composed of one TSR domain and five galactose-binding lectin domains (Fig 2B). XM_002160899 mRNA was strongly expressed in the epithelial cells of the ectoderm in the tentacles and hypostome (Figs 1F and 3H), though the mRNA of this gene was sparsely detected in the body column (Fig 1F).

The third group was marked by the concanavalin A-like lectin/glucanase domain with a single TSR domain (Fig 2C). Two genes (XM_002154891 and XM_002163692) were identified as members of this family. The cDNA clones for XM_002154891 mRNA were the second most abundant among all clones obtained in this study (56 clones, Table 1), and were strongly detected in the non-epithelial cells of the endoderm in the hypostome, possibly gland mucous cells (Fig 3I). In contrast, the other gene in this family, XM_002163692 was expressed in the epithelial cells of the ectoderm in the hypostome (Fig 3J).

**Fig 4:**
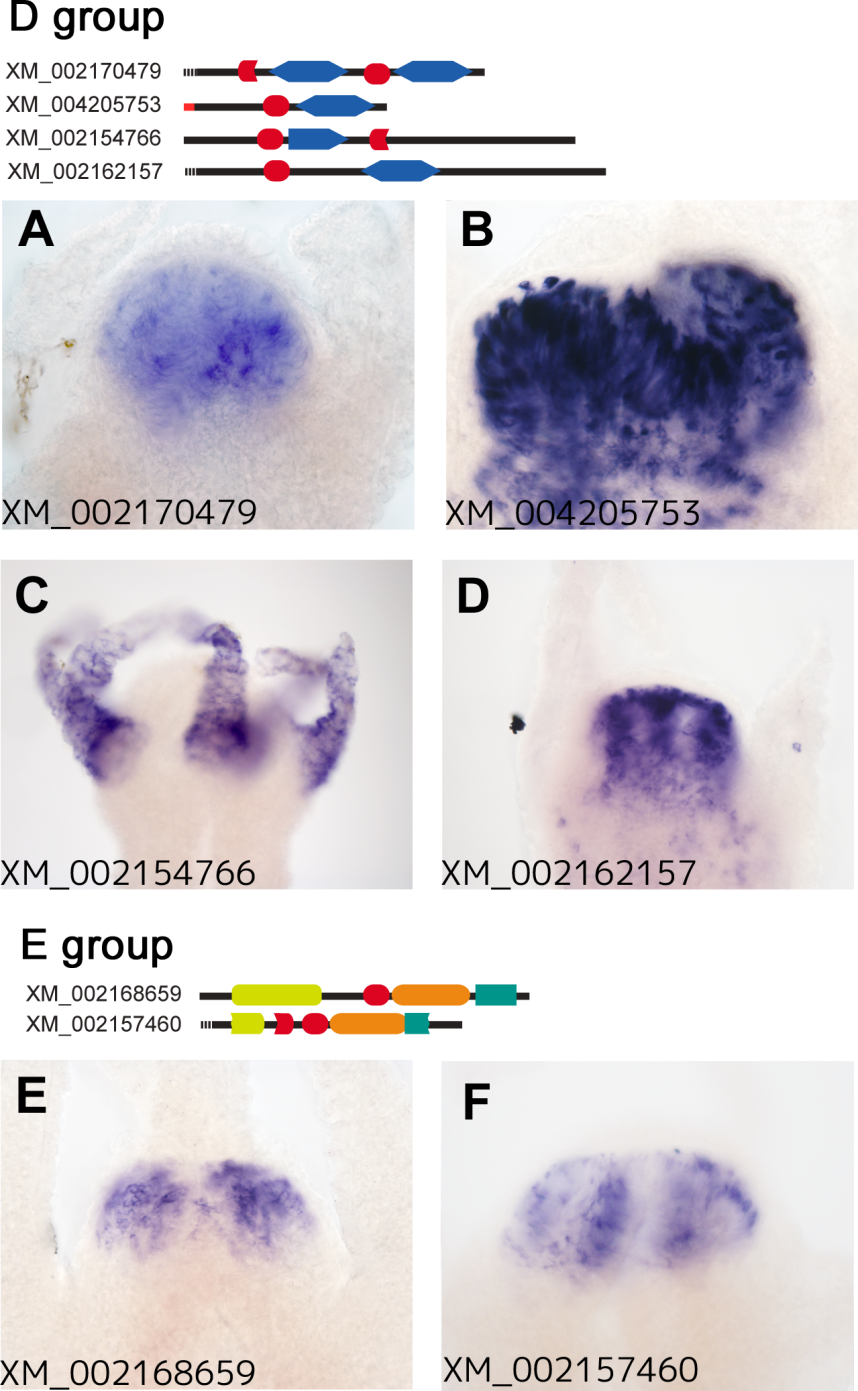
Expression patterns of the genes encoding TSR domain proteins of the D and E groups in the head region of *Hydra oligactis*. Among the 6 genes of the D and E groups, five (A, B, D-F) are strongly expressed in the endodermal cells of the head region. The transcript of XM_002154766 (C) was only detected in the tentacles, and not in the hypostome. Accession numbers were labeled on the lower left of each panel. The domain structures of the genes were shown on the top of the photo panels for each group.

The fourth group comprised four genes (XM_002170479, XM_004205753, XM_002154766, and XM_002162157), which had one or two TSR domains with the von Willebrand factor type A (vWA) domain in common (Fig 2D). The vWA domain is considered to act as an interaction module in a number of proteins [36]. The mRNAs of three members of this family, XM_002170479, XM_004205753, and XM_002162157, were expressed in the epithelial cells of the endoderm in the hypostome (Fig 4A, 4B and 4D). In contrast, that of the other member, XM_002154766, was detected in the epithelial cells of the ectoderm in the tentacles (Fig 4C).

The fifth group was classified by the existence of the zinc-dependent metalloprotease domain. Although it comprised three genes (XM_002168659, XM_002157460, and XM_002166845), the domain structure of XM_002166845 appeared to markedly differ from those of the other members, which share the cell-wall integrity and stress-response component 1 (WSC) domain and Meprin, A5 protein, and protein tyrosine phosphatase Mu (MAM) domain (Fig 2E). XM_002168659 and XM_002157460 mRNAs were both detected in the non-epithelial cells of the endoderm in the hypostome using our WISH procedure (Fig 4E and 4F), whereas the expression of XM_002166845 was not.

## Discussion

We examined genes selectively expressed in the head region of *Hydra oligactis* using SSH. Among these genes, we identified 17 that encoded TSR domain proteins. Of the cDNA clones obtained in the present study, 25.3% were derived from genes encoding TSR domain proteins. Our WISH analyses confirmed that eight of the 17 genes we identified were exclusively expressed in the hypostome, five were expressed in the other regions as well as in the hypostome, and three were expressed only in the tentacles (Figs 1, 3 and 4 and S1 Fig).

As suggested by the results of WISH analyses, our SSH procedure may effectively concentrate cDNA clones for head-specific genes. Notably, multiple cDNA clones for *Hydra Wnts* and *HyBra2*, a putative Wnt downstream gene, were found in the SSH cDNA library. These genes have been reported to be intensely expressed in the hypostome of *Hydra magnipapillata* (Wnts) [10] and *Hydra vulgaris* (HyBra2) [33]. Furthermore, we found multiple cDNA clones for Frizzled, a component of Wnt receptors, in the SSH library. It will be important to confirm whether Frizzled is expressed in the head region of *Hydra* by WISH.

The TSR domain, which consists of ~60 amino acid residues, was initially detected in the thrombospondin-1 (THBS1) protein in human platelets. THBS1 is a multifunctional matrix glycoprotein that plays roles in platelet aggregation, angiogenesis, and tumorigenesis [37]. TSR domains in THBS1 have been shown to mediate binding to extracellular matrix components such as collagen, fibronectin, laminin, integrin, glycosaminoglycan chains, and von Willebrand factor [38].

TSR domain proteins have been identified in various species from protozoa to vertebrates. In *Hydra*, we identified 185 TSR domain proteins in the NCBI Protein database (http://www.ncbi.nlm.nih.gov/protein). Thus, the TSR domain proteins identified in the present study account for a small proportion (9.2%) of all Hydra TSR domain proteins.

The TSR domain proteins in the present study were tentatively classified into five groups according to the domain combinations. In order to determine whether these domain combinations are evolutionary conserved, we used the Conserved Domain Architecture Retrieval Tool (CDART) [39] at the NCBI site, and found that the first group characterized by multiple TSR domains with no other predicted domains and the second group marked by the existence of the galactose-binding lectin domain with TSR domains appeared to be cnidarian-specific domain combinations. TSR domain proteins in these groups may function in cnidarian-specific biological process.

Most of the domain structures of TSR domain proteins shown here are predicted from the genomic and cDNA sequences of *Hydra magnipapillata*, the genome of which has been sequenced and analyzed. Thus, we cannot rule out the possibility that the *bona fide* domain structures of these TSR domain proteins are different from those shown here. Moreover, lack of the information about the genomic and cDNA sequences of *Hydra oligactis* did not allow us to directly determine the domain structures of the TSR domain proteins in *Hydra oligactis*. Further studies to determine full-length cDNA sequences for the subtracted clones encoding the TSR domain proteins are required.

The TSR domain has been identified within a number of proteins including R-spondin, F-spondin, SCO-spondin, Semaphorin-5A (Sema5A), UNC-5, complement factors, the TRAP proteins of plasmodium, and the ADAMTS family [28, 29]. These TSR superfamily proteins are generally involved in the regulation of cell-cell or cell-matrix interaction processes such as cell migration, cell adhesion, and axon guidance. R-spondin family proteins have been shown to potentiate Wnt signaling [40, 41], which is involved in many different aspects of development. Since the Wnt signaling pathway in *Hydra* plays important roles in establishing and maintaining the head organizer [4, 9], some of the TSR domain proteins we isolated in the present study may modulate the Wnt pathway and affect the development of the organizer.

Several TSR domain proteins, such as Sema5A, F-spondin, SCO-spondin, TSP-1 and UNC-5, are known to be involved in axonal guidance and growth in the vertebrate nervous system [28, 29]. The TSR domains of Sema5A interact with the glycosaminoglycan portion of chondroitin sulfate proteoglycans and heparan sulfate proteoglycans, and play important roles in axonal guidance in the development of the fasciculus retroflexus in mice [42]. Thus, any of the TSR domain-containing proteins isolated in the present study may be involved in the formation or maintenance of the neural network in the hypostome of *Hydra*.

Another possible function of the TSR domain proteins in *Hydra* may be in defense against pathogens. The most frequently cloned gene, *Tsrp2* (AM233513), encodes a rhamnospondin (Rsp)-like protein [43]. Rsp genes were originally isolated in the colonial hydroid *Hydractinia symbiolongicarpus* (Phylum Cnidaria) and are predicted to encode multi-domain secretory proteins containing a rhamnose-binding lectin domain and eight TSR domains [44]. The Rsp genes were shown to be specifically and constitutively expressed in the hypostomes of gastrozooid mouths and the gene products have been proposed to bind to microorganisms entering into the gastrovascular cavity. The microorganisms with Rsp proteins may then be removed by phagocytosis, or by purging through the mouth after forming aggregates [44]. In *Hydra*, the *Tsrp2* gene may act in a similar manner to Rsp.

## Supporting Information

S1 Fig Expression patterns of the genes encoding TSR domain proteins in *Hydra oligactis* excluding those shown in Fig 1. Expression patterns of the 5 genes in A group (A-E), the 4 genes in D group (F-I) and the 2 genes in E group (J and K). Accession numbers were labeled under each panel. The domain structures of the genes were shown on the top of photo panels for A group, and the right side for D and E group.
(PDF)

S1 Table Results of a BLAST homology search of cDNA clones isolated from the SSH library of *Hydra oligactis*. This table is continued from Table 1.
(DOCX)
