# Supplementary figures and images for: Thrombospondin Type-1 Repeat Domain-Containing Proteins Are Strongly Expressed in the Head Region of Hydra
Source: PLoS One. 2016 Apr 4;11(4):e0151823. doi: 10.1371/journal.pone.0151823 (PMC4820225; doi:10.1371/journal.pone.0151823)

## A group

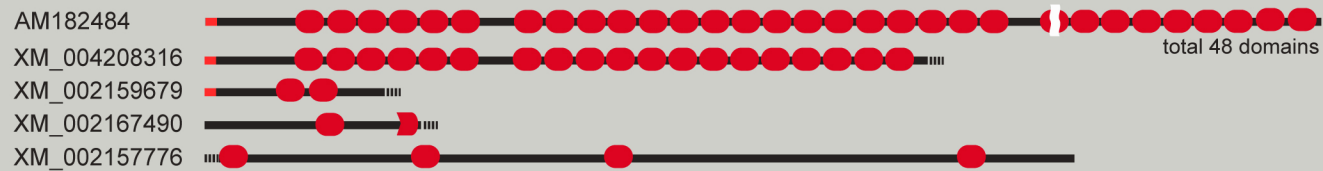

A

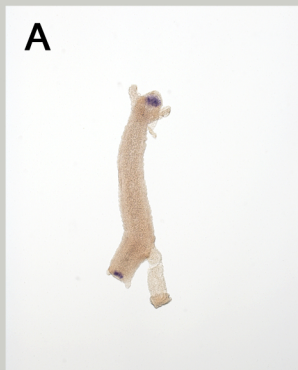

AM182484

B

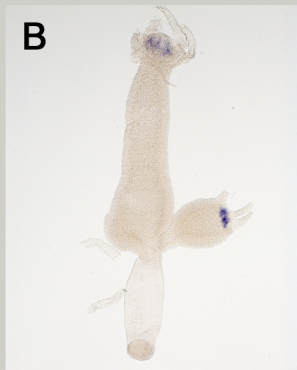

XM\_004208316

C

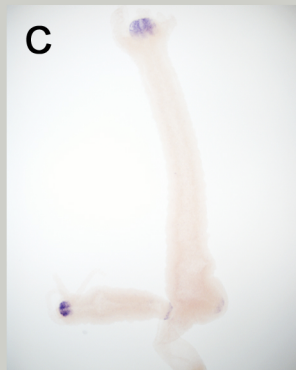

XM\_002159679

D

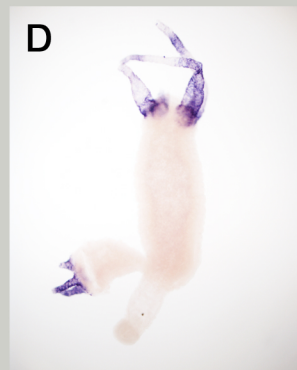

XM\_002167490

E

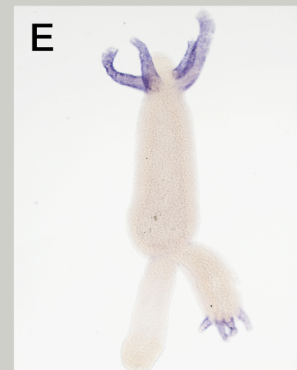

XM\_002157776

F

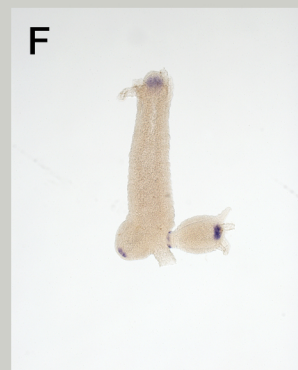

XM\_002170479

G

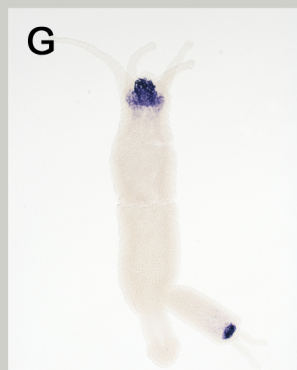

XM\_004205753

H

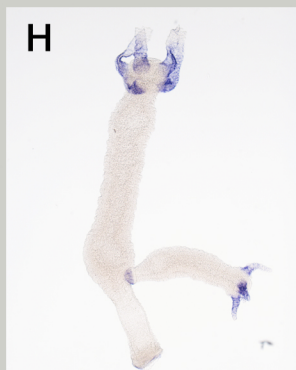

XM\_002154766

I

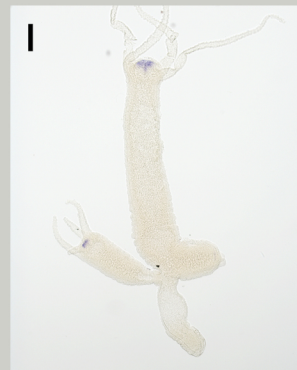

XM\_002162157

## D group

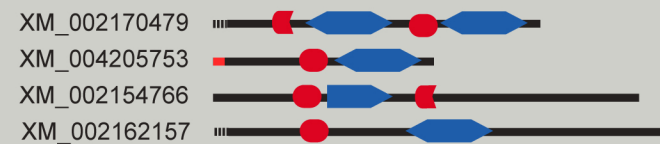

J

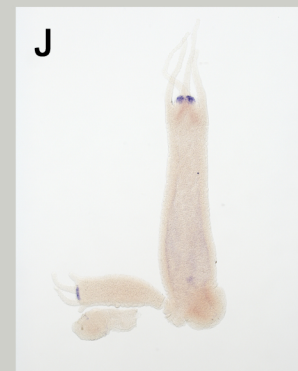

XM\_002168659

K

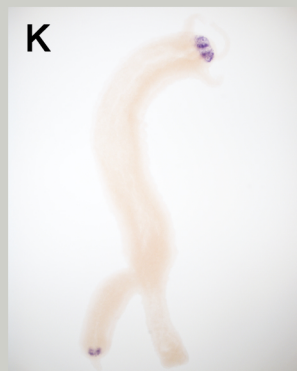

XM\_002157460

## E group

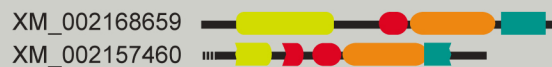

Supplement: S1 Fig — Expression patterns of the 5 genes in A group (A-E), the 4 genes in D group (F-I) and the 2 genes in E group (J and K). Accession numbers were labeled under each panel. The domain structures of the genes were shown on the top of photo panels for A group, and the right side for D and E group. (PDF) [file pone.0151823.s001.pdf]
