# Supplementary material for: Thrombospondin Type-1 Repeat Domain-Containing Proteins Are Strongly Expressed in the Head Region of Hydra
Source: PLoS One. 2016 Apr 4;11(4):e0151823. doi: 10.1371/journal.pone.0151823 (PMC4820225; doi:10.1371/journal.pone.0151823)
Supplement: S1 Table — This table is continued from Table 1. (DOCX) [file pone.0151823.s002.docx]

**S1 Table. Results of a BLAST homology search of cDNA clones isolated from the SSH library of *Hydra oligactis*.** This table is continued from Table 1.

| **N** | **Gene name (best BLASTN match  with E-value <1e-10)** | | **Accession  number ^a^** | | **Number  of clones ^b^** | **E-value ^c^** |
| --- | --- | --- | --- | --- | --- | --- |
| 37 | *Hydra vulgaris* 28S large subunit ribosomal RNA gene, partial sequence | | EU879941 | | 3 | 0.0.E+00 |
| 38 | *Hydra vulgaris* transcription factor ZNF436 mRNA, complete cds | | JQ994210 | | 3 | 4.1.E-19 |
| 39 | PREDICTED: *Hydra magnipapillata* uncharacterized LOC100210883 (LOC100210883), partial mRNA | | XM_002153746 | | 3 | 0.0.E+00 |
| 40 | PREDICTED: *Hydra magnipapillata* transcription factor 12-like (LOC100202254), mRNA | | XM_002153870 | | 3 | 4.7.E-180 |
| 41 | PREDICTED: *Hydra magnipapillata* secreted signaling factor Wnt7 (hywnt7), mRNA | | XM_002153912 | | 3 | 3.3.E-110 |
| 42 | PREDICTED: *Hydra magnipapillata* myosin heavy chain, striated muscle-like (LOC100205834), partial mRNA | | XM_002157890 | | 3 | 0.0.E+00 |
| 43 | PREDICTED: *Hydra magnipapillata* uncharacterized LOC100201149 (LOC100201149), mRNA | | XM_002158562 | | 3 | 0.0.E+00 |
| 44 | PREDICTED: *Hydra magnipapillata* tubulin beta chain-like (LOC100199191), mRNA | | XM_002161824 | | 3 | 8.6.E-78 |
| 45 | PREDICTED: *Hydra magnipapillata* kinesin-like protein KLP6-like (LOC100209483), mRNA | | XM_002161855 | | 3 | 8.8.E-124 |
| 46 | PREDICTED: *Hydra magnipapillata* uncharacterized LOC100213474 (LOC100213474), mRNA | | XM_002162498 | | 3 | 2.4.E-81 |
| 47 | PREDICTED: *Hydra magnipapillata* uncharacterized LOC100215743 (LOC100215743), mRNA | | XM_002163737 | | 3 | 0.0.E+00 |
| 48 | PREDICTED: *Hydra magnipapillata* 72 kDa type IV collagenase-like (LOC100199972), mRNA | | XM_002163912 | | 3 | 6.8.E-154 |
| 49 | PREDICTED: *Hydra magnipapillata* protein PRY1-like (LOC100200105), mRNA | | XM_002164010 | | 3 | 8.9.E-22 |
| 50 | PREDICTED: *Hydra magnipapillata* uncharacterized LOC100205082 (LOC100205082), mRNA | | XM_002164648 | | 3 | 2.1.E-78 |
| 51 | PREDICTED: *Hydra magnipapillata* fibrillin-2-like (LOC100202765), mRNA | | XM_002166838 | | 3 | 7.5.E-79 |
| **52** | **PREDICTED: *Hydra magnipapillata* uncharacterized LOC100210942 (LOC100210942), partial mRNA** | | **XM_002167490** | | **3** | **2.6.E-134** |
| 53 | PREDICTED: *Hydra magnipapillata* 26S proteasome non-ATPase regulatory subunit 1-like (LOC100199064), partial mRNA | | XM_002168098 | | 3 | 2.8.E-163 |
| 54 | PREDICTED: *Hydra magnipapillata* laminin subunit alpha-like (LOC100200618), partial mRNA | | XM_002170337 | | 3 | 5.6.E-134 |
| 55 | PREDICTED: *Hydra magnipapillata* elongation factor 1-alpha-like (LOC100206683), mRNA | | XM_004205474 | | 3 | 2.8.E-47 |
| **56** | ***Hydra vulgaris* mRNA for HyTSR1 protein, strain Basel** | | **AM182484** | | **2** | **3.3.E-98** |
| 57 | *Schmidtea mediterranea* clone HB.2.11g unknown mRNA sequence | | AY066159 | | 2 | 2.0.E-17 |
| 58 | *Schistosoma japonicum* isolate Anhui full-length mRNA clone SJFCE3850.027\|FSE001-P00026-M18, complete sequence | | FN322610 | | 2 | 9.4.E-21 |
| 59 | Cloning vector pCST5-del-SAT1, complete sequence | | HQ536210 | | 2 | 0.0.E+00 |
| 60 | PREDICTED: *Hydra magnipapillata* talin 2 (tln2), mRNA | | XM_002154489 | | 2 | 0.0.E+00 |
| 61 | PREDICTED: *Hydra magnipapillata* innexin inx1-like (LOC100197789), mRNA | | XM_002154760 | | 2 | 0.0.E+00 |
| 62 | PREDICTED: *Hydra magnipapillata* major vault protein-like (LOC100209402), mRNA | | XM_002154832 | | 2 | 0.0.E+00 |
| 63 | PREDICTED: *Hydra magnipapillata* ATP-binding cassette sub-family D member 4-like (LOC100208446), mRNA | | XM_002155849 | | 2 | 0.0.E+00 |
| 64 | PREDICTED: *Hydra magnipapillata* uncharacterized LOC100209787 (LOC100209787), mRNA | | XM_002155903 | | 2 | 0.0.E+00 |
| 65 | PREDICTED: *Hydra magnipapillata* uncharacterized LOC100211098 (LOC100211098), mRNA | | XM_002155937 | | 2 | 1.6.E-60 |
| 66 | PREDICTED: *Hydra magnipapillata* type I inositol 3,4-bisphosphate 4-phosphatase-like (LOC100202677), mRNA | | XM_002157013 | | 2 | 5.7.E-134 |
| 67 | PREDICTED: *Hydra magnipapillata* uncharacterized LOC100206447 (LOC100206447), mRNA | | XM_002157064 | | 2 | 3.1.E-43 |
| 68 | PREDICTED: *Hydra magnipapillata* elongation factor 2-like (LOC100203564), mRNA | | XM_002157752 | | 2 | 0.0.E+00 |
| **69** | **PREDICTED: *Hydra magnipapillata* SCO-spondin-like (LOC100197287), partial mRNA** | | **XM_002157776** | | **2** | **1.2.E-58** |
| 70 | PREDICTED: *Hydra magnipapillata* tropomodulin-3-like (LOC100213797), partial mRNA | | XM_002157839 | | 2 | 2.3.E-178 |
| 71 | PREDICTED: *Hydra magnipapillata* homeobox protein OTX2-B-like (LOC100207886), partial mRNA | | XM_002158995 | | 2 | 6.1.E-79 |
| 72 | PREDICTED: *Hydra magnipapillata* E3 ubiquitin-protein ligase PDZRN3-like (LOC100199004), mRNA | | XM_002159038 | | 2 | 1.8.E-102 |
| 73 | PREDICTED: *Hydra magnipapillata* elongation of very long chain fatty acid protein 5-like (LOC100210039), mRNA | | XM_002159380 | | 2 | 0.0.E+00 |
| 74 | PREDICTED: *Hydra magnipapillata* 15-hydroxyprostaglandin dehydrogenase [NAD(+)]-like (LOC100212103), mRNA | | XM_002159646 | | 2 | 5.9.E-103 |
| 75 | PREDICTED: *Hydra magnipapillata* zinc metalloproteinase nas-15-like (LOC100208103), mRNA | | XM_002159944 | | 2 | 1.1.E-14 |
| 76 | PREDICTED: *Hydra magnipapillata* uncharacterized LOC100205548 (LOC100205548), mRNA | | XM_002160289 | | 2 | 0.0.E+00 |
| 77 | PREDICTED: *Hydra magnipapillata* potassium voltage-gated channel subfamily C member 3-like (LOC100206086), partial mRNA | | XM_002160890 | | 2 | 2.2.E-82 |
| 78 | PREDICTED: *Hydra magnipapillata* titin-like (LOC100212532), partial mRNA | | XM_002161034 | | 2 | 0.0.E+00 |
| 79 | PREDICTED: *Hydra magnipapillata* uncharacterized LOC100215593 (LOC100215593), mRNA | | XM_002161128 | | 2 | 4.6.E-103 |
| 80 | PREDICTED: *Hydra magnipapillata* UDP-N-acetylglucosamine--peptide N-acetylglucosaminyltransferase 110 kDa subunit-like (LOC100201589), mRNA | | XM_002161466 | | 2 | 0.0.E+00 |
| 81 | PREDICTED: *Hydra magnipapillata* BTB/POZ domain-containing protein KCTD16-like (LOC100210833), mRNA | | XM_002162020 | | 2 | 5.1.E-130 |
| 82 | PREDICTED: *Hydra magnipapillata* transmembrane protease serine 6-like (LOC100214705), mRNA | | XM_002162525 | | 2 | 1.3.E-111 |
| 83 | PREDICTED: *Hydra magnipapillata* Frizzled4/9/10 (hyfzd4/9/10), mRNA | | XM_002163078 | | 2 | 4.9.E-72 |
| 84 | PREDICTED: *Hydra magnipapillata* cadherin EGF LAG seven-pass G-type receptor 2-like (LOC100215197), partial mRNA | | XM_002163205 | | 2 | 4.7.E-37 |
| 85 | PREDICTED: *Hydra magnipapillata* integrin alpha-5-like (LOC100208443), mRNA | | XM_002163535 | | 2 | 2.9.E-82 |
| 86 | PREDICTED: *Hydra magnipapillata* eukaryotic translation initiation factor 3 subunit D-like (LOC100202564), mRNA | | XM_002164100 | | 2 | 4.5.E-148 |
| 87 | PREDICTED: *Hydra magnipapillata* uncharacterized LOC100213152 (LOC100213152), mRNA | | XM_002165440 | | 2 | 7.3.E-104 |
| 88 | PREDICTED: *Hydra magnipapillata* pre-mRNA-splicing factor syf2-like (LOC100214439), mRNA | | XM_002165572 | | 2 | 2.6.E-53 |
| 89 | PREDICTED: *Hydra magnipapillata* proteasome subunit alpha type-2-like (LOC100209532), mRNA | | XM_002166160 | | 2 | 0.0.E+00 |
| 90 | PREDICTED: *Hydra magnipapillata* cyclin-dependent kinase-like 1-like (LOC100202809), partial mRNA | | XM_002166252 | | 2 | 2.8.E-165 |
| 91 | PREDICTED: *Hydra magnipapillata* 60S ribosomal protein L7-like (LOC100208095), mRNA | | XM_002166304 | | 2 | 5.2.E-161 |
| 92 | PREDICTED: *Hydra magnipapillata* uncharacterized LOC100200796 (LOC100200796), mRNA | | XM_002166561 | | 2 | 1.8.E-122 |
| 93 | PREDICTED: *Hydra magnipapillata* glutamine synthetase-like (LOC100212131), mRNA | | XM_002167112 | | 2 | 3.7.E-48 |
| 94 | PREDICTED: *Hydra magnipapillata* uncharacterized LOC100212286 (LOC100212286), mRNA | | XM_002167623 | | 2 | 2.2.E-180 |
| 95 | PREDICTED: *Hydra magnipapillata* uncharacterized LOC100197480 (LOC100197480), mRNA | | XM_002167834 | | 2 | 2.2.E-63 |
| 96 | PREDICTED: *Hydra magnipapillata* uncharacterized LOC100209722 (LOC100209722), partial mRNA | | XM_002168254 | | 2 | 0.0.E+00 |
| 97 | PREDICTED: *Hydra magnipapillata* uncharacterized LOC100208925 (LOC100208925), mRNA | | XM_002168437 | | 2 | 0.0.E+00 |
| 98 | PREDICTED: *Hydra magnipapillata* tropomodulin-2-like (LOC100213204), mRNA | | XM_002168510 | | 2 | 1.4.E-47 |
| 99 | PREDICTED: *Hydra magnipapillata* uncharacterized LOC100207471 (LOC100207471), mRNA | | XM_002168666 | | 2 | 6.7.E-72 |
| 100 | PREDICTED: *Hydra magnipapillata* uncharacterized LOC100205584 (LOC100205584), partial mRNA | | XM_002169889 | | 2 | 3.3.E-112 |
| 101 | PREDICTED: *Hydra magnipapillata* uncharacterized LOC100205787 (LOC100205787), mRNA | | XM_002170381 | | 2 | 9.9.E-17 |
| 102 | PREDICTED: *Hydra magnipapillata* cadherin EGF LAG seven-pass G-type receptor 1-like (LOC100201710), mRNA | | XM_002170684 | | 2 | 4.6.E-85 |
| 103 | PREDICTED: *Hydra magnipapillata* protein FAM132A-like (LOC101241396), mRNA | | XM_004206345 | | 2 | 1.1.E-126 |
| 104 | PREDICTED: *Hydra magnipapillata* cpG-binding protein-like (LOC100200520), mRNA | | XM_004207208 | | 2 | 1.2.E-150 |
| 105 | PREDICTED: *Hydra magnipapillata* trace amine-associated receptor 7b-like (LOC101237956), mRNA | | XM_004208323 | | 2 | 4.6.E-31 |
| 106 | PREDICTED: *Hydra magnipapillata* uncharacterized LOC101236954 (LOC101236954), mRNA | | XM_004209029 | | 2 | 3.8.E-146 |
| 107 | PREDICTED: *Hydra magnipapillata* adenylate kinase isoenzyme 5-like (LOC100215094), mRNA | | XM_004209988 | | 2 | 0.0.E+00 |
| 108 | PREDICTED: *Hydra magnipapillata* uncharacterized LOC101234511 (LOC101234511), partial mRNA | | XM_004211022 | | 2 | 6.3.E-37 |
| 109 | PREDICTED: *Hydra magnipapillata* nascent polypeptide-associated complex subunit alpha, muscle-specific form-like, transcript variant 2 (LOC100209087), mRNA | | XM_004212289 | | 2 | 0.0.E+00 |
| 110 | PREDICTED: *Hydra magnipapillata* cyclin-dependent kinase 17-like (LOC101240338), partial mRNA | | XM_004212860 | | 2 | 8.6.E-93 |
| 111 | PREDICTED: *Hydra magnipapillata* peptidyl-prolyl cis-trans isomerase-like 4-like (LOC100200683), misc_RNA | | XR_053556 | | 2 | 0.0.E+00 |
| 112 | PREDICTED: *Hydra magnipapillata* uncharacterized LOC100215630 (LOC100215630), partial misc_RNA | | XR_181934 | | 2 | 7.1.E-34 |
| ^a^ Accession numbers were annotated according to the NCBI database. | | | | |  |  |
| ^b^ The number of sequenced clones in the SSH library. | | |  |  |  |  |
| ^c^ The best e-value from a BLASTN search. | | |  |  |  |  |
